# Oxidized protein aggregate lipofuscin impairs cardiomyocyte contractility via late-stage autophagy inhibition

**DOI:** 10.1016/j.redox.2025.103559

**Published:** 2025-02-19

**Authors:** Sophia Walter, Steffen P. Häseli, Patricia Baumgarten, Stefanie Deubel, Tobias Jung, Annika Höhn, Christiane Ott, Tilman Grune

**Affiliations:** a Molecular Toxicology, German Institute of Human Nutrition Potsdam-Rehbruecke (DIfE), Nuthetal, Germany; b TraceAge-DFG Research Unit on Interactions of Essential Trace Elements in Healthy and Diseased Elderly, Potsdam-Berlin-Jena-Wuppertal, Germany; c DZHK (German Center for Cardiovascular Research), Partner Site Berlin, Berlin, Germany; d Institute of Nutritional Science, Department of Food Chemistry, University of Potsdam, Potsdam, 14469, Germany; e German Center for Diabetes Research (DZD), München-Neuherberg, Germany; f Cardiac Aging and Nutrition, German Institute of Human Nutrition Potsdam-Rehbruecke (DIfE), Nuthetal, Germany

**Keywords:** Heart, Lysosome, Contraction, Aging

## Abstract

Aging of the heart is accompanied by impairment of cardiac structure and function. At molecular level, autophagy plays a crucial role in preserving cardiac health. Autophagy maintains cellular homeostasis by facilitating balanced degradation of cytoplasmic components including organelles and misfolded or aggregated proteins. The age-related decline in autophagy favors an accumulation of protein aggregates such as lipofuscin particularly in the heart, which is composed primarily of non-proliferating cells. Therefore, this study investigates whether lipofuscin accumulation contributes to age-related functional decline of primary adult cardiomyocytes isolated from C57BL/6J mice and examines the role of autophagic flux in mediating these effects.

Results showed an age-associated reduction in cardiomyocyte contraction amplitude and an increase in autofluorescence, indicating the accumulation of lipofuscin with age. *In vitro* treatment of adult primary cardiomyocytes with artificial lipofuscin increased autofluorescence and decreased both contraction amplitude and cellular autophagic flux. Induction of autophagy with rapamycin mitigated contractile dysfunction in lipofuscin-treated cardiomyocytes, whereas inhibition of autophagic flux revealed stage-dependent effects. Late-stage autophagy inhibition using chloroquine or concanamycin A reduced cardiomyocyte contraction amplitude, whereas early-stage autophagy inhibition via 3-methyladenine did not affect contraction within 24 h.

In conclusion, our results indicate that lipofuscin directly impairs cardiomyocyte function by diminishing late-stage autophagic flux. These findings highlight the essential role of the autophagy-lysosomal system in preserving age-related loss of cardiomyocyte function caused by accumulating protein aggregates.

## Graphical abstract

**Figure:**
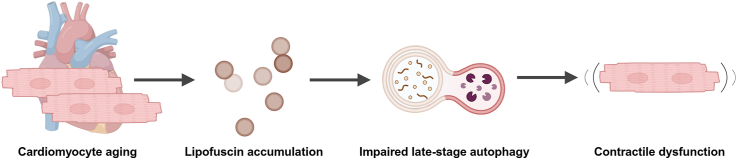

## 1 Introduction

Aging of the heart is associated with structural and functional changes observed as increase in left-ventricular mass, fibrosis and contractile dysfunction [1]. A growing body of evidence indicates that a key factor contributing to cardiac aging is the impairment of autophagy [2]. Macroautophagy (here: autophagy) describes the formation of a double membrane vesicle, the autophagosome, which sequesters cytosolic cargo for lysosomal degradation [3]. Loss of cardiac autophagy in autophagy-related gene (Atg) 5 knockout mice was associated with a shortened lifespan accompanied by systolic and diastolic dysfunction [4], whereas overexpression of Atg5 increased lifespan and attenuated age-related cardiac fibrosis [5].

The process of autophagy starts with the initiation by phosphorylation of the Unc-51 Like Autophagy Activating Kinase 1 (ULK-1) complex, which is predominantly induced by nutrient starvation. The following step, nucleation or elongation, is mediated by class III phosphoinositide 3-kinase (PI3K) complex leading to production of phosphatidylinositol 3-phosphate (PI3P), whose downstream proteins recruit Atg9 membrane-containing vesicles and the Atg5-Atg12-Atg16L1 complex. Thereafter, the elongated autophagic membrane encloses and the resulting autophagosome fuses with the lysosome to form the autolysosome, in which encased substrates are degraded by lysosomal hydrolases [3,6]. To measure autophagy, the use of autophagy inhibitors is mandatory. Inhibition of autophagic flux enables the detection of accumulating autophagy marker proteins [7]. These proteins include microtubule-associated protein light chain 3 (LC3) and sequestome-1 (p62). During nucleation, LC3-I, the unconjugated form, can be converted to LC3-II through conjugation to phosphatidylethanolamines at the autophagosome membrane. P62 binds to LC3 and ubiquitinated substrates, which enables the degradation of targeted substrates [3]. Inhibitors of autophagic flux are used at different stages. For example, 3-methyladenine (3-MA) is applied as an early-stage autophagy inhibitor, since it inhibits class I and III PI3K [8,9]. Chloroquine (CQ) suppresses autophagosome-lysosome fusion and increases lysosomal pH [10], whereas concanamycin A (ConA) blocks lysosomal vacuolar-type ATPases (vATPase), which also deacidifies the lysosomes [11], making both, CQ and ConA, late-stage autophagy inhibitors.

Disruption of autophagy and increase in oxidative stress, both present during aging, are associated with accumulation of oxidized and aggregated proteins including lipofuscin [12]. Lipofuscin accumulates in aged tissue predominately consisting of postmitotic cells, such as eye, brain but also heart muscle [13]. It even correlates with chronological age in human hearts making it a valuable marker to estimate human age [14]. Artificial lipofuscin consists of oxidized proteins, lipids, sugars and small amounts of transition metals [12], recently, we have shown that this also applies to human heart lipofuscin [15]. Intracellularly, lipofuscin is primarily found in lysosomes due to its lysosomal origin or its sequestration via autophagy [16]. Because of its highly cross-linked structure, lipofuscin cannot be degraded. On the contrary, it is able to diminish proteasomal and lysosomal function [15,17,18]. Thus, lipofuscin-loaded cells are particularly prone to oxidative stress [17] and senescence [19], emphasizing the role of lipofuscin in mediating aging and associated diseases.

Therefore, our aim was to understand the contribution of lipofuscin to age-related decline in cardiomyocyte contractility and to analyze the role of autophagy in this context. Previous studies showed that aging is associated with elevated levels of cardiac lipofuscin [14,19,20], decreased autophagy [4,19,21] and impairment of cardiomyocyte function [[22], [23], [24]], however, the interplay of these factors in cardiac aging has not yet been proven.

## 2 Materials and methods

### 2.1 Animal models

Male C57BL/6JRj mice were obtained from Janvier Lab and bred in-house. Experiments were conducted in young mice with 5–8 months of age. Old mice were sacrificed with 24 months of age. Mice were housed at 40–50 % humidity and 22 ± 2 °C under 12 h light/dark cycle and received a standard rodent chow (V1534, Ssniff Spezialdiäten GmbH) and water *ad libitum*. Experiments and animal housing were carried out according to German law on protection of animals. Since mice were sacrificed for organ removal, no further ethical approval was required (§4 Abs. 3 TierschG).

### 2.2 Lipofuscin preparation

Artificial lipofuscin was produced from human erythrocyte membranes as described before [25]. Lysed erythrocytes were homogenized in hypotonic PBS. Using centrifugation and washing circles, the membrane fraction was purified. Protein content was measured by Bradford and adjusted to 3 g/l. It was assumed that protein content before irradiation equals the amount of lipofuscin after irradiation. UV irradiation (100 J/cm^2^ UVA and 50 J/cm^2^ UVB) was applied in 100 mm petri dishes containing 30 ml of the membrane fraction. Autofluorescence of the membrane fraction was measured at different excitation/emission pairs (360 nm excitation/460 nm emission, 360 nm excitation/590 nm emission, 360 nm excitation/460 nm emission) until a plateau was reached.

### 2.3 Cardiomyocyte isolation

Cardiomyocyte isolation was performed based on the Langendorff-Free Method [26] as described earlier [27]. In brief, mice were anesthetized by isoflurane followed by sacrifice via cervical dislocation. The heart was removed and rinsed with EDTA and perfusion buffer and subsequently enzymatically and mechanically digested. The cell suspension was filtered by a 100 μm pore-size strainer. Gravity settings and calcium reintroduction were performed in 3 rounds.

### 2.4 Cardiomyocyte cell culture

Isolated cardiomyocytes were dissolved in plating medium and added to laminin-coated plates (5 μg/ml). For all analyses the same culture medium was used composed of M199 medium (41150020, ThermoFisher), 0.1 % BSA, 1x insulin-transferrin-sodium selenite (I3146, Sigma), 10 mM 2,3-butanedione monoxime, 1x chemically defined lipid concentrate (11905, Gibco), and penicillin-streptomycin (P06-07100, PAN biotech). Lipofuscin was used in concentrations of 0.025–0.1 mg/ml in PBS for up to 18 h. PBS was used as the solvent control. Samples for mRNA measurement were treated for 6 h. Rapamycin (R8781, Sigma) was solved in DMSO, the solution was applied with a final concentration of 100 nM in medium for 18 h, a DMSO control was applied in the same way. Concanamycin A (ConA) dissolved in DMSO (C9705, Merck) was prediluted in medium to a stock concentration of 5 μM and further diluted in medium 1:1000 to a final concentration of 5 nM. Chloroquine (CQ; Sigma, C6628) was dissolved in H_2_O to a stock concentration of 10 mM, filtered and further diluted to a final concentration of 10 μM in medium. A H_2_O control was used in the same proportion. 3-MA (M9281, Sigma) was dissolved in prewarmed medium and the filtered solution was used at a final concentration of 5 mM. Since 3-MA and ConA were directly compared to each other, DMSO was also applied in 3-MA sample and control. Cells were treated with inhibitors for 24 h unless otherwise indicated. After incubation, cells for protein and mRNA detection were washed twice with PBS containing 10 mM of contraction inhibitor 2,3-butanedione monoxime and stored at −80 °C.

### 2.5 Western Blot

To harvest cells, SDS lysis buffer [10 mM Tris (pH = 7.5), 0.9 % NP-40, 0.1 % SDS, 1 mM Pefabloc®] containing protease inhibitor (11873580001, Roche) was added to the cardiomyocytes. Protein concentration was determined using Lowry assay. Samples were diluted in Laemmli buffer [0.25 mM Tris (pH = 6.8), 40 % glycerol, 20 % 2-mercaptoethanol, 8 % SDS, 0.03 % Bromophenol Blue] and boiled for 5 min at 95 °C. Proteins were separated by SDS polyacrylamide gel electrophoresis and transferred to nitrocellulose membranes (10600001, Cytiva). Membranes were blocked for 1 h at RT and primary antibodies were applied overnight at 4 °C. Used primary antibodies included p62 (ab56416, abcam), LC3A/B (12741, Cell signaling) and glyceraldehyde-3-phosphate dehydrogenase (GAPDH, ab8245, abcam). After washing, secondary antibodies (962–68022 and 925–32211, LI-COR) were applied for 1 h at RT. Washed membranes were scanned by the Odyssey® CLx Imaging System (LI-COR). Images of the membranes were analyzed using Image Studio Software (v 5.2, LI-COR). GAPDH was used as housekeeping protein.

To calculate the autophagic flux, the signal of p62 or LC3-II was divided by the respective GAPDH signal. The normalized p62 and LC3-II values of inhibitor-treated samples were then divided by their respective control without inhibitor treatment. The following equation shows an example of the calculation of the autophagic flux with p62 as an example protein and ConA as an inhibitor:

$$Autophagic\;flux\;\left(x-fold\;induction\right)=\frac{\left(\frac{{p62}_{ConA}}{{GAPDH}_{ConA}}\right)}{\left(\frac{{p62}_{Ctrl}}{{GAPDH}_{Ctrl}}\right)}$$

### 2.6 Real-time PCR

Isolation of mRNA was performed using the Dynabead™ mRNA DIRECT™ kit (61012, Invitrogen) according to the protocol of the manufacturer. For cDNA synthesis SeniFAST™ cDNA Synthesis Kit (65054, Bioline Reagents) was used. Samples were diluted 1:10 in nuclease-free water. qPCR analyses were performed with 1 μl DreamTaq™ buffer, 0.05 μl DreamTaq™ Hot Start DNA-Polymerase (EP1702, ThermoFisher), 2 mM dNTPs (39028, Bioline Reagents), 1x SYBR™ Green (S7563), 1.25 μM forward and reverse primer. The heating cycle started with 3 min of 95 °C, followed by 40 cycles of denaturation for 15 s at 95 °C, primer hybridization for 30 s at 60 °C and elongation for 30 s at 72 °C. The following primer pairs were used: LC3 (forward: 5′-GACCAGCACCCCAGTAAGAT-3′, reverse: 5′-T GGGACCAGAAACTTGGTCT-3′) and p62 (forward: 5′-AGATGCCAGAATCGGAAGGG-3′, reverse: 5′-GAGAGGGACTCAATCAGCCG-3′). The genes β-Actin (forward: 5′-CACTGCCGCATCCTCTTCCT-3′, reverse: 5′-GATTCCATACCCAAGAAGGAAGGC-3′), GAPDH (forward: 5′-GGGTGTGAACCACGAGAAAT-3′, reverse: 5′-GTCTTCTGGGTGGCAGTGAT-3′), hypo-xanthine phosphoribosyl transferase 1 (forward: 5′-GCAGTCCCAGCGTCGTG-3′, reverse: 5′-GGCCTCCCATCTCCTTCAT-3′), and ribosomal protein L13a (forward: 5′-GTTCGGCTGAAGCCTACCAG-3′, reverse:5′-TTCCGTAACCTCAAGATCTGCT-3′) were taken as housekeeping genes. All primers were obtained from Sigma-Aldrich. Standard curves of amplified cDNA were used to calculate the amount of sample cDNA with help of MxPro qPCR Software (Agilent Technologies). All housekeeping genes were used to calculate a normalization factor and amount of target cDNA was divided by this normalization factor.

### 2.7 Autofluorescence

For autofluorescence measurement, live-cell imaging of isolated cardiomyocytes, seeded in laminin-precoated glass bottom dishes, was performed. Images were taken with the LSM 780 confocal microscope using a 40× LD Plan-Neofluar objective (Zeiss). Autofluorescence was determined with an excitation wavelength of 405 nm and an emission wavelength of 498 nm ± 20 nm. At least 20 cells were measured per sample and animal. Autofluorescence intensity per cell subtracted by the estimated background was analyzed using a macro in ImageJ.

### 2.8 Cardiomyocyte contraction analysis

For contraction measurement directly after cell isolation, warm Krebs-Ringer solution (137 mM NaCl, 5.4 mM KCl, 0.5 mM MgSO_4_, 10 mM d-glucose, 1 mM CaCl_2_∗2H_2_O, 0.4 mM K_2_HPO_4_∗3H_2_O, 25 mM NaHCO_3_; pH = 7.4) was added to the cells after 1 h of plating and contraction was immediately measured with the Calcium and Contraction System from IonOptix. Contraction measurement after lipofuscin incubation was performed in prewarmed Krebs-Ringer solution and contraction measurement after inhibitor treatment was performed in prewarmed Tyrode buffer (137 mM NaCl, 5.4 mM KCl, 0.5 mM MgSO_4_, 10 mM Glucose, 1 mM CaCl_2_∗2H_2_O, 11.8 mM Hepes; pH = 7.4). Prewarmed buffer was pumped through the system to ensure optimal and constant temperature during measurement. Cell contraction parameter were measured during stimulating with 10 V at 1 Hz. At least 10 cells per sample and animal were analyzed, each of which showed at least 10 reliable contraction transients. Data was obtained and analyzed using the program Cytosolver received from IonOptix. Results showed changes in sarcomere length per time (Suppl. Fig. 1).

### 2.9 Statistics

Statistical analyses were performed using GraphPad Prism (v. 9.5.0). Outliers were removed as indicated by the Rout Outlier test (Q = 1 %). Normality was tested using Shapiro Wilk test. Induction to control was calculated by dividing all samples by the mean of the controls. Due to signal variations between the membranes (Fig. 4), the induction per animal was calculated by dividing each treatment by the respective control. Unpaired *t*-test (normality passed) or Mann-Whitney *U* test (normality not passed) were performed to test for significance with only 2 groups. Data with 3 or more groups was analyzed with One-way ANOVA (normality passed). To test for significant induction per animal One-sample *t*-test (normality passed) or One-sample Wilcoxon test (normality not passed) were used with a hypothetical mean of 1. Results are presented as mean ± SD and statistical significance was considered at p ≤ 0.05.

## 3 Results

### 3.1 Effect of aging on cardiomyocyte function and autofluorescence

**Fig. 1:**
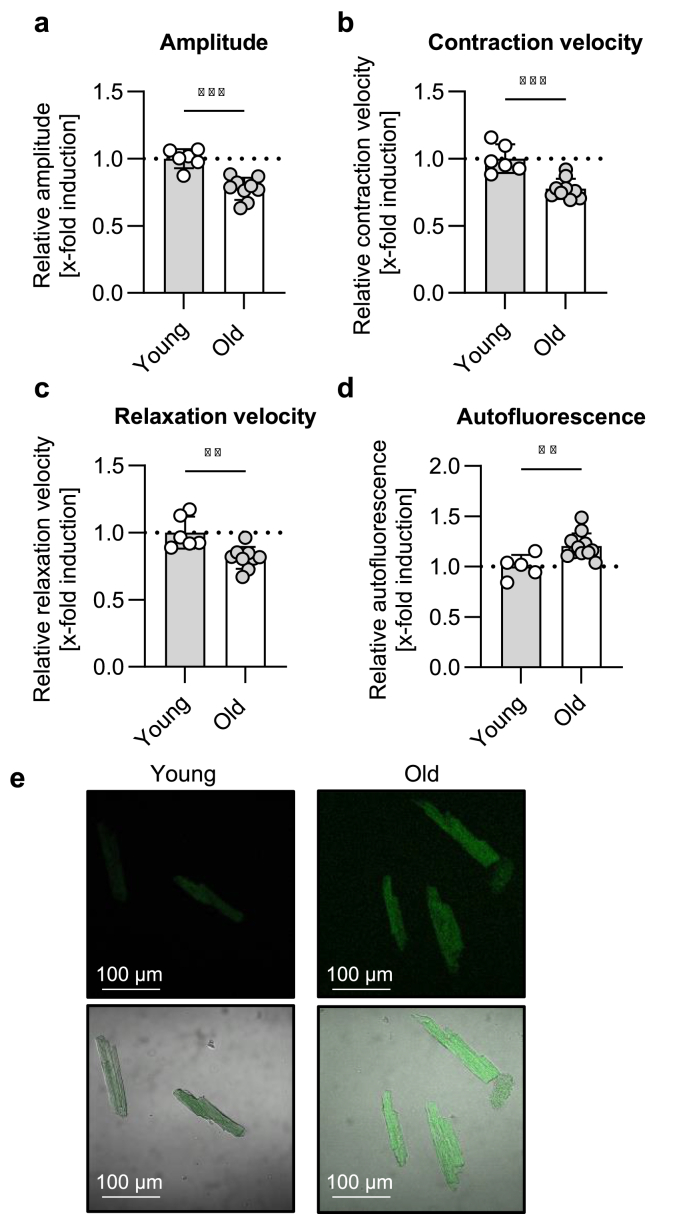
**Aging is associated with reduced contraction and increased autofluorescence in murine cardiomyocytes. (a**–**c)** Contraction was measured in freshly isolated cardiomyocytes from young (5–8 month) and old (24 month) mice. The relative amplitude (percentage of shortening, %) as well as relative contraction and relaxation velocity (μm/sec) were determined. **(d**–**e)** Relative average autofluorescence intensity per cell (a.u.) was analyzed via live-cell imaging using excitation of 405 nm and emission of 498 nm. Data represent mean ± SD. Statistical significance was tested with students t-test and given as follows: ∗∗p ≤ 0.01, ∗∗∗p ≤ 0.001.

To assess potential differences in cardiomyocyte function, contraction was measured in freshly isolated cells from young (5–8 month) and old (24 month) mice. Cardiomyocytes from old mice showed a lower contraction amplitude as well as a reduced contraction and relaxation velocity compared to cells isolated from young mice (Fig. 1a–c). In addition to contractile dysfunction, previous studies have shown that cardiac aging is associated with accumulation of protein aggregates such as lipofuscin [14]. To verify the presence of protein aggregates in our model, we performed live-cell imaging of isolated cardiomyocytes from young and old mice and evaluated autofluorescence at 405 nm wavelength excitation and 498 nm emission, which is a common marker for determination of protein aggregates [15,25]. Results proofed higher autofluorescence in cardiomyocytes isolated from old compared to young mice, indicating a higher amount of lipofuscin in old cardiomyocytes (Fig. 1d–e).

### 3.2 Role of lipofuscin for cardiomyocyte contraction

**Fig. 2:**
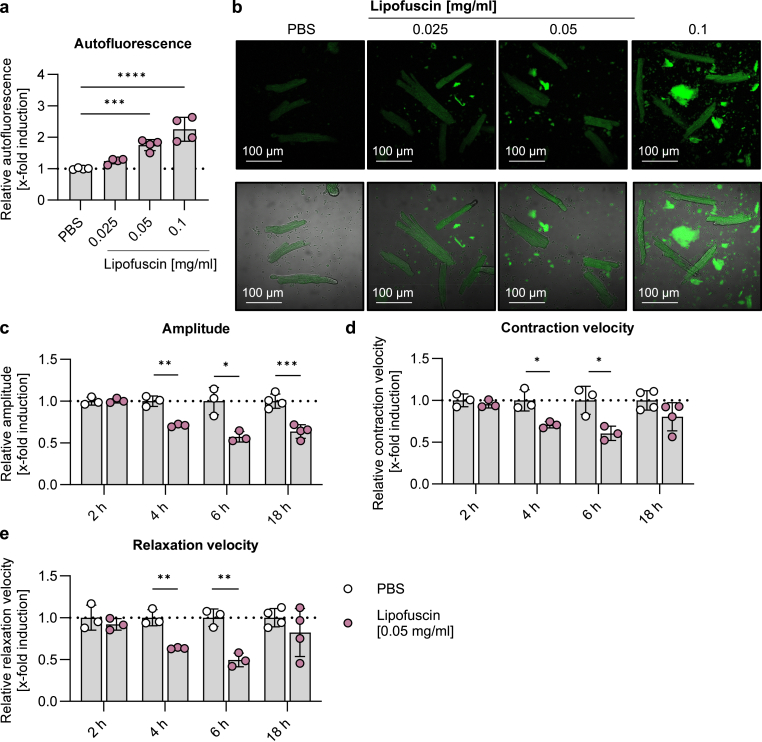
**Treatment of cardiomyocytes with lipofuscin decreases cardiomyocyte contraction. (a**–**b)** Cardiomyocytes from young mice were treated with artificial lipofuscin for 18 h and relative autofluorescence (a.u.) was analyzed in living cells using excitation of 405 nm and emission of 498 nm. PBS was used as a respective control. **(c**–**e)** Cardiomyocytes were treated for 2–18 h with 0.05 mg/ml artificial lipofuscin and contraction was measured. The relative amplitude (percentage of shortening, %) as well as relative contraction and relaxation velocity (μm/sec) were determined. Data represent mean ± SD. Statistical significance was tested with **(a)** One-way ANOVA or **(c**–**e)** students t-test comparing PBS and lipofuscin and given as follows: ∗p ≤ 0.05, ∗∗p ≤ 0.01, ∗∗∗p ≤ 0.001, ∗∗∗∗p ≤ 0.0001.

Cardiomyocytes isolated from young mice were exposed for 18 h with artificial lipofuscin solved in PBS in a concentration range from 0.025 to 0.1 mg/ml. With increasing lipofuscin concentration, cardiomyocytes exhibited increasing autofluorescence intensity (Fig. 2a–b). We decided to use 0.05 mg/ml lipofuscin in the following experiments, as treatment with 0.05 mg/ml lipofuscin for 18 h did not affect viability of adult cardiomyocytes (Suppl. Fig. 2), but still showed a significant increase in autofluorescence. Accordingly, young cardiomyocytes were treated with 0.05 mg/ml lipofuscin for 2, 4, 6 and 18 h. Cardiomyocyte contraction amplitude was reduced after 4 h and 6 h, this effect persisted in the 18 h treatment (Fig. 2c). Contraction and relaxation velocities were reduced after 4 h and 6 h, whereas this effect was not present after the 18 h treatment (Fig. 2d–e). To take potential cultivation effects into account, we compared contraction of primary cardiomyocytes 4 h and 6 h after isolation with cardiomyocytes 18 h after isolation. Results revealed a significant lower contraction and relaxation velocity in older cells as cultivation increased time to peak and time to baseline 10 % without affecting contraction amplitude (Suppl. Fig. 3).

### 3.3 Link between lipofuscin and cardiomyocyte autophagic flux

**Fig. 3:**
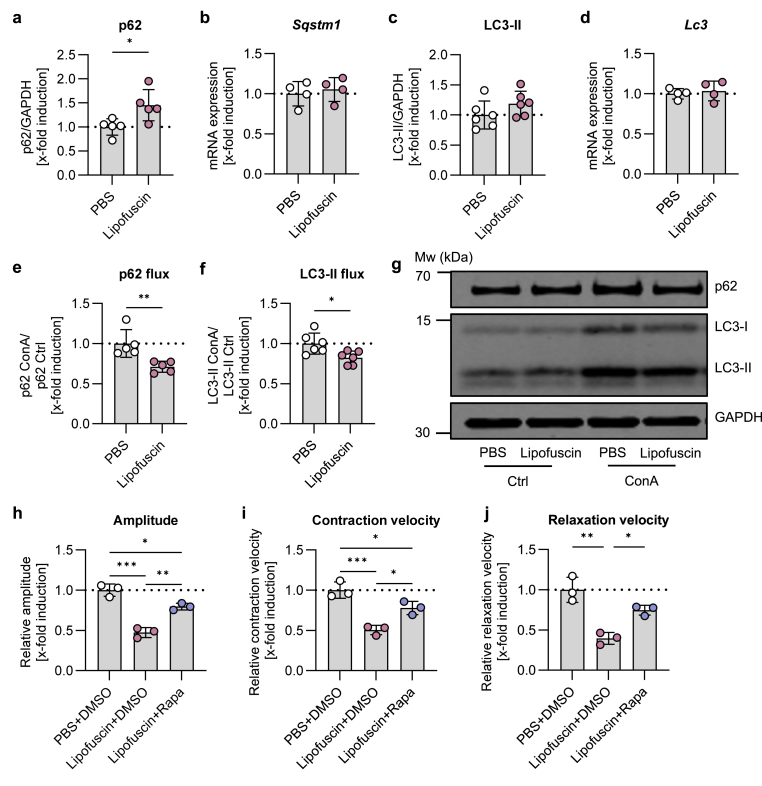
**Lipofuscin diminishes autophagic flux and lipofuscin-associated cardiomyocyte dysfunction is attenuated by rapamycin-induced autophagy. (a**–**d)** Cardiomyocytes from young mice were treated with 0.05 mg/ml artificial lipofuscin, PBS was used as a respective control. **(a,c)** Basal normalized protein levels of p62 and LC3-II after 24 h treatment with lipofuscin were analyzed by immunoblot. **(b,d)** Expression of mRNA of Sqstm1/p62 and Lc3 after 6 h treatment with lipofuscin were measured using qPCR. **(e**–**f)** For autophagic flux measurement, concanamycin A (ConA) was used as an inhibitor in presence or absence of lipofuscin for 24 h. Normalized protein levels of p62 and LC3-II with ConA were divided by the protein levels without ConA to calculate the autophagic flux. **(h**–**j)** Cardiomyocyte function was measured after 18 h treatment with lipofuscin in presence or absence of autophagic flux inducer rapamycin (Rapa). The relative amplitude (percentage of shortening, %) as well as relative contraction and relaxation velocity (μm/sec) were determined. Data represent mean ± SD. Statistical significance was tested with **(a**–**f)** students t-test or **(h**–**j)** One-way ANOVA and given as follows: ∗p ≤ 0.05, ∗∗p ≤ 0.01, ∗∗∗p ≤ 0.001. Mw: molecular weight.

Lipofuscin is known to affect proteolytic function [15,17,18]. Especially autophagy is used for cytosolic clearance from highly-crosslinked protein aggregates, which cannot be unfolded (aggrephagy) [28]. Accordingly, we measured the effect of lipofuscin treatment on cardiomyocyte autophagy-related proteins. Because a 6 h treatment with lipofuscin was not sufficient to change basal p62 protein levels (Suppl. Fig. 4), cardiomyocytes were treated for 24 h with 0.05 mg/ml lipofuscin. Lipofuscin treatment increased basal p62 protein levels without affecting Sqstm1/p62 mRNA levels in cardiomyocytes (Fig. 3a–b), indicating accumulation of autophagic receptor p62. Basal LC3-II protein levels and Lc3 mRNA were not affected by lipofuscin treatment (Fig. 3c–d). Additionally, we used ConA as a lysosomal inhibitor in a co-treatment with lipofuscin to investigate the autophagic flux during a period when lipofuscin alters basal autophagy (see methods section for autophagic flux calculation). The ConA-related accumulation of p62 and LC3-II were reduced in lipofuscin-treated cardiomyocytes compared to control cardiomyocytes (Fig. 3e–g). Thereby our results indicate, that lipofuscin caused a reduced autophagic flux in cardiomyocytes. To investigate whether lipofuscin-mediated decrease of autophagic flux is associated with declining cardiomyocyte contractility and to demonstrate a link between both processes, we co-treated cardiomyocytes with lipofuscin and the autophagy inducer rapamycin. Again, lipofuscin decreased contraction amplitude as well as contraction and relaxation velocities. Interestingly, rapamycin significantly improved the lipofuscin-related reduction of contraction parameters, although it did not completely restore cardiomyocyte function after 18 h treatment (Fig. 3h–j).

**Fig. 4:**
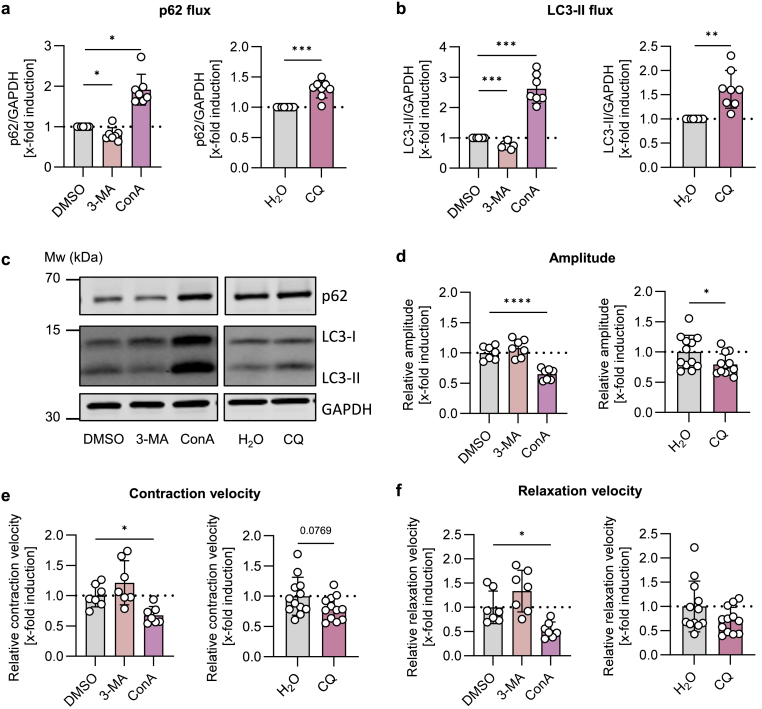
**Late-stage not early-stage autophagic flux inhibition impairs cardiomyocyte contraction within 24 h.** Cardiomyocytes isolated from young mice were treated for 24 h with autophagic flux inhibitors 3-methyladenine (3-MA), chloroquine (CQ) and concanamycin A (ConA). **(a**–**c)** The effects on autophagic flux protein and **(d**–**f)** cardiomyocyte contractility were measured. The relative amplitude (percentage of shortening, %) as well as relative contraction and relaxation velocity (μm/sec) were determined. Data represent mean ± SD. Statistical significance was tested with **(a**–**b)** one sample *t*-test (normality passed) or one sample Wilcoxon test (normality not passed) or **(d**–**f)** One-way ANOVA (DMSO, 3-MA, ConA) or students t-test (normality passed)/Mann-Whitney *U* test (normality not passed) (H_2_O and CQ) and given as follows: ∗p ≤ 0.05, ∗∗p ≤ 0.01, ∗∗∗p ≤ 0.001, ∗∗∗∗p ≤ 0.0001. Mw: molecular weight.

### 3.4 Effect of autophagic flux inhibition on cardiomyocyte function

As we were able to show that lipofuscin-mediated impairment of cardiomyocyte contraction was associated with a decreased autophagic flux, we next analyzed whether a reduced autophagic flux is able to impair adult cardiomyocyte function directly. Accordingly, we treated cardiomyocytes isolated from young mice with three different autophagy inhibitors for 24 h because the effects of autophagy inhibitors should be compared to the effects of lipofuscin. 3-MA was used as an early-stage inhibitor, while CQ and ConA were used as late-stage inhibitors. Additionally, previous experiments revealed that short-time treatment (2 h) with autophagic flux inhibitor CQ similar to lipofuscin was not long enough to affect cardiomyocyte contraction (Suppl. Fig. 5). The inhibitory effect on autophagic flux was proofed on protein level. ConA and CQ increased p62 and LC3-II protein levels, whereas 3-MA decreased p62 and LC3-II (Fig. 4a–c). LC3-I was elevated by 3-MA and ConA incubation but not by CQ treatment (Suppl. Fig. 6). On functional level, ConA and CQ decreased cardiomyocyte contraction amplitude, with ConA showing the strongest effect (Fig. 4d). Additionally, ConA reduced contraction and relaxation velocity and CQ showed a trend towards a decreased contraction velocity (p = 0.08, Fig. 4e–f).

## 4 Discussion

Our study revealed that aging is associated with functional impairment of cardiomyocytes and accumulation of autofluorescent protein aggregates, such as lipofuscin. In young cells, artificial lipofuscin treatment caused contractile dysfunction and impaired autophagic flux. A combined treatment of lipofuscin and rapamycin attenuated the lipofuscin-associated reduction in cardiomyocyte contraction. Experiments using autophagic flux inhibitors revealed that late-stage autophagic inhibition decreased cardiomyocyte contraction comparable to lipofuscin treatment, suggesting that aging-related protein aggregates impair late-stage autophagy, and thereby contributing to the functional decline of primary cardiomyocytes.

In agreement with our results, the majority of mouse studies have demonstrated that aging is associated with contractile dysfunction in cardiomyocytes, which can be measured as a diminished cardiomyocyte contraction amplitude [29]. In addition to declined cardiomyocyte function, we pointed out a rise in cellular autofluorescence, indicating accumulation of fluorescent protein aggregates such as lipofuscin with age [30]. Age-related accumulation of lipofuscin in cardiomyocytes and whole heart tissue was obtained in various physiological and *in vitro* aging models throughout the years [14,19,20,31].

To investigate the role of lipofuscin for cardiomyocyte performance, young cells were treated with lipofuscin *in vitro*. We used artificial lipofuscin produced from human erythrocyte membranes, which has been shown to be a good alternative to the authentic lipofuscin due to its similar emission peak and matching of other characteristics [25]. Interestingly, 2 h exposure with artificial lipofuscin did not affect cardiomyocyte function, whereas longer exposure times (4, 6 and 18 h) caused a detrimental reduction in contraction amplitude. Monitoring autofluorescence after lipofuscin treatment in fibroblasts revelated a progressive uptake of lipofuscin, leading to a 2.75-fold increase in autofluorescence after 72 h [18]. Accordingly, we suggest, that lipofuscin did not induce an acute toxic effect but its gradual uptake diminished contractile function in primary cardiomyocytes. However, 18 h treatment with lipofuscin did not change contraction nor relaxation velocity, whereas 4 h and 6 h lipofuscin treatment decreased velocities. We figured out that these different effects were confounded by the impact of 18 h cultivation on primary adult cardiomyocytes, as cultivation increased time to peak and time to baseline. Accordingly, when interpreting the results, it has to be kept in mind that adult cardiomyocytes can be affected by longer cultivation time, which in our study was visible from 18 h onwards.

In addition to cardiomyocyte contractility, lipofuscin was shown to affect the autophagic flux. Previous studies reported contradictory results in lipofuscin-rich cells and tissues, with autophagy markers being reduced [19], unchanged [14] or even increased [16,32,33] compared to cells and tissues with lower lipofuscin content. We revealed higher basal p62 protein levels upon lipofuscin treatment, while p62 mRNA, Lc3 mRNA and basal LC3-II protein were not affected. Because of the increase in p62 protein levels, we suggest that lipofuscin triggers accumulation of ubiquitinated proteins prone for degradation via the autophagy lysosomal system [34]. To investigate the autophagic flux in lipofuscin-treated cells, we used the lysosomal inhibitor ConA in parallel with lipofuscin. Lipofuscin-treated cells exhibited a reduced turnover of p62 and LC3-II upon 24 h ConA treatment compared to control cells, which proved that lipofuscin decreased autophagic flux in young cardiomyocytes. One reason for this reduced autophagic flux can be the ability of lipofuscin to disrupt lysosomal function [15,17]. The suspected lysosomal impairment can lead to a reduced autophagic flux by limiting the degradation of autophagosomes, autolysosomes and their substrates [35], which we pointed out in our cell model. Nevertheless, the autophagic flux itself is crucial for lysosomal accumulation and degradation of lipofuscin [16,36]. Knockout of Atg5 reduced lysosomal lipofuscin and increased cytosolic lipofuscin, which is more detrimental for cellular survival than lysosomal lipofuscin [16]. Thus, autophagy is essential for lipofuscin detoxification. However, lipofuscin itself is able to impair the autophagy lysosomal system [15,16], indicating a vicious circle of accumulating protein aggregates.

To counteract this vicious circle in our cells, we used rapamycin to enhance autophagy in lipofuscin-treated cardiomyocytes. Addition of rapamycin mitigated the contractile dysfunction of lipofuscin-treated cardiomyocytes, resulting in a higher contraction amplitude as well as contraction and relaxation velocity compared to lipofuscin-treated cells. Our results underline the role of autophagy in lipofuscin-associated disruption of cardiomyocyte function. However, rapamycin treatment was not able to completely restore the effect of lipofuscin on contraction within 18 h. Potentially, lipofuscin caused an irreversible damage of cardiomyocyte function and/or autophagy. In addition, reduced autophagy may not be the only mechanism by which lipofuscin decreases cardiomyocyte function, because lipofuscin can also impair e.g. proteasomal function [18]. Moreover, increasing autophagy at the very early stage of the autophagic machinery, by suppression of mTORC1 using rapamycin [37], may not completely restore lipofuscin-mediated decrease of late-stage autophagy. In accordance, we hypothesize, that lipofuscin reduced cardiomyocyte contractility by decreasing late-stage autophagic flux and lysosomal function.

The relevance of autophagy for cardiac health was shown in various animal models. For example, cardiac Atg5 knockout mice develop a cardiomyopathy characterized by hypertrophy, fibrosis and reduced fractional shortening, compared to wild-type mice, leading to a low median life span of 1 year [4]. Furthermore, elevated autophagy induced via a whole-body knock-in of beclin-1 prevented the origin of age-related fibrosis and cardiomyocyte hypertrophy in mice [38]. In this study we used 3 different autophagic flux inhibitors, 3-MA, ConA and CQ, and investigated their effects on cardiomyocyte function. 3-MA, an early autophagic flux inhibitor, is known to inhibit class I and III PI3K. Inhibition of class III PI3K enzymes blocks the production of PI3P and subsequently the recruitment of Atg proteins to the autophagic membrane [8]. Previously it was shown that 3-MA can have a dual role on the autophagic flux, as it can also increase autophagy in nutrient-rich medium when used in long-term treatment (>9 h) measured as a prolonged conversion of LC3-I to LC3-II [9]. However, we showed reduced LC3-II and increased LC3-I protein levels in cardiomyocytes treated for 24 h with 3-MA, indicating a reduction of the autophagic flux at an early stage. Nevertheless, inhibition of the autophagic flux using 3-MA did not affect cardiomyocyte contraction amplitude nor velocities, which corresponds to the results of previous studies [39,40]. In contrast to cell culture experiments using 3-MA, a cardiac knockout of class III PI3K reduced contractility in whole hearts [41], suggesting that extended decrease of early autophagic flux is able to diminish cardiac function.

Inhibitors ConA and CQ were used to block late-stage autophagic flux. ConA deacidifies lysosomes by inhibiting vATPases, leading to accumulation of autophagosomes and dysfunctional lysosomes [11,42]. In our experiments, ConA reduced contraction amplitude as well as contraction and relaxation velocity in young cardiomyocytes. Interestingly, accumulation of autophagic vacuoles in hearts of mice with a lysosomal associated membrane protein (Lamp) 2 knockout increased mortality and decreased contractile function [43], underlining the role of functional lysosomes for cardiac health. Moreover, lysosomal inhibition can increase autophagosome formation via reducing mTORC1 activation [7]. In ConA-treated cardiomyocytes, LC3-I protein levels were enhanced after 24 h, suggesting compensatory autophagosome formation, further leading to autophagosome accumulation as evidenced by increased LC3-II level, potentially contributing to its detrimental effect on contraction. In contrast to ConA, CQ treatment decreased cardiomyocyte contraction amplitude but did not significantly affect contraction nor relaxation velocity. CQ is able to act directly on cardiomyocyte contraction by blocking voltage-dependent Na^+^, Ca^2+^ and K^+^ channels and decreasing Ca^2+^ influx [44]. In this study we used CQ to address the direct effect of late-stage autophagic flux, especially the fusion of autophagosomes and lysosomes [10]. Inhibition of autophagosome-lysosome fusion in human induced pluripotent stem cell-derived cardiomyocytes with Lamp2B knockout resulted in contractile impairment of these cells, highlighting the importance of the fusion process for cardiomyocyte function [45].

Results revealed, that only pharmacological inhibition of late-stage autophagy detrimentally reduced cardiomyocyte contraction. One possible factor contributing to the contractile impairment may be an abnormal Ca^2+^ signaling. Cardiomyocytes from mice with a disrupted Lamp2 gene, associated with accumulation of immature lysosomes and autophagosome, showed lower contraction and relaxation time due to disturbed Ca^2+^ signaling [46]. On molecular level, lysosomal deacidification can trigger Ca^2+^ efflux from lysosomes into the cytosol [47]. Due to the presence of a lysosome-sarcoplasmic reticulum Ca^2+^ axis [48,49], we hypothesized, that changed lysosomal Ca^2+^ levels as induced by lysosomal deacidification using ConA and CQ, may disturb Ca^2+^ signaling in our model. Moreover, failing of autophagy, as induced by CQ, causes an increase in oxidative stress [50], which in turn can cause redox modulation of Ca^2+^ handling proteins, which affects their activity and subsequently cardiomyocyte Ca^2+^ signaling [51,52].

In addition to the potential role of Ca^2+^, Redmann et al. (2017) revealed another possible molecular mechanism by which the use of these inhibitors can disrupt cellular function. The authors showed that bafilomycin A1 and CQ treatment impaired mitochondrial function and quality in primary neurons by reduced activity of mitochondrial enzymes and increased mitochondrial damage, without affecting mitochondrial DNA [53]. These findings indicate, that mitochondrial function may be diminished by autophagic inhibition. The combination of disrupted autophagic recycling and diminished mitochondrial function may result in an energy deficit, leading to contractile dysfunction in cardiomyocytes treated with lipofuscin or autophagy inhibitors.

## 5 Conclusion

Lipofuscin accumulation with age is associated with impaired contraction in murine cardiomyocytes. In cells isolated from young mice, lipofuscin decreased contraction amplitude and disrupted autophagic flux. Notably, only the inhibition of late-stage autophagic flux decreased cardiomyocyte contraction amplitude, accordingly to lipofuscin treatment. Therefore, results indicate a link between age-related accumulation of lipofuscin and late-stage autophagy impairment which triggered contractile dysfunction. Previous data on lipofuscin and autophagy in the heart indicate that aging and protein aggregates such as lipofuscin reduce autophagy in cardiomyocytes and that an improvement in autophagy by rapamycin alleviates lipofuscin accumulation in the heart [19]. Furthermore, we were able to show that rapamycin-induced autophagy attenuates the contractile dysfunction of cardiomyocytes caused by lipofuscin. Consequently, a moderate increase of autophagy in lipofuscin-loaded tissue is able to counteract age-associated effects such as senescence and contractile dysfunction. Therefore, induction of physiological autophagic flux in aging tissue may represent a promising strategy to combat lipofuscin-driven cardiac decline to rejuvenate the aging heart and preserve cardiac health over time.

## CRediT authorship contribution statement

**Sophia Walter:** Writing – original draft, Visualization, Methodology, Investigation, Formal analysis. **Steffen P. Häseli:** Writing – review & editing, Methodology, Investigation, Formal analysis. **Patricia Baumgarten:** Writing – review & editing, Investigation. **Stefanie Deubel:** Writing – review & editing, Investigation. **Tobias Jung:** Writing – review & editing, Software. **Annika Höhn:** Writing – review & editing, Methodology, Investigation. **Christiane Ott:** Writing – review & editing, Validation, Supervision, Funding acquisition, Data curation, Conceptualization. **Tilman Grune:** Writing – review & editing, Supervision, Resources, Funding acquisition.

## Data availability statement

The data that support the findings of this study are available from the corresponding author upon reasonable request.

## Funding

TG was supported by DFG-funded research group TraceAge (FOR 2558). CO and TG are funded by the 10.13039/100010447DZHK (German Center for Cardiovascular Research; 81Z2100502). TG is also funded by the 10.13039/501100001659Deutsche Forschungsgemeinschaft (10.13039/501100001659DFG, German Research Foundation; Gr1240/25–1) and TG and AH are supported by DZD (German Center for Diabetes Research; Grant 82DZD0034G). AH is funded by the 10.13039/501100001659Deutsche Forschungsgemeinschaft (10.13039/501100001659DFG, German Research Foundation; HO 5332/4–1).

## Declaration of competing interest

None.

## Data Availability

Data will be made available on request.

## References

[1] Dai D.-F., Chen T., Johnson S.C., Szeto H., Rabinovitch P.S. (2012). Cardiac aging: from molecular mechanisms to significance in human health and disease. Antioxidants Redox Signal..

[2] Abdellatif M., Sedej S., Carmona-Gutierrez D., Madeo F., Kroemer G. (2018). Autophagy in cardiovascular aging. Circ. Res..

[3] Dikic I., Elazar Z. (2018). Mechanism and medical implications of mammalian autophagy. Nat. Rev. Mol. Cell Biol..

[4] Taneike M., Yamaguchi O., Nakai A., Hikoso S., Takeda T., Mizote I. (2010). Inhibition of autophagy in the heart induces age-related cardiomyopathy. Autophagy.

[5] Pyo J.-O., Yoo S.-M., Ahn H.-H., Nah J., Hong S.-H., Kam T.-I. (2013). Overexpression of Atg5 in mice activates autophagy and extends lifespan. Nat. Commun..

[6] Yamamoto H., Zhang S., Mizushima N. (2023). Autophagy genes in biology and disease. Nat. Rev. Genet..

[7] Klionsky D.J., Abdel-Aziz A.K., Abdelfatah S., Abdellatif M., Abdoli A., Abel S. (2021). Guidelines for the use and interpretation of assays for monitoring autophagy (4th edition)1. Autophagy.

[8] Petiot A., Ogier-Denis E., Blommaart E.F., Meijer A.J., Codogno P. (2000). Distinct classes of phosphatidylinositol 3'-kinases are involved in signaling pathways that control macroautophagy in HT-29 cells. J. Biol. Chem..

[9] Wu Y.-T., Tan H.-L., Shui G., Bauvy C., Huang Q., Wenk M.R. (2010). Dual role of 3-methyladenine in modulation of autophagy via different temporal patterns of inhibition on class I and III phosphoinositide 3-kinase. J. Biol. Chem..

[10] Mauthe M., Orhon I., Rocchi C., Zhou X., Luhr M., Hijlkema K.-J. (2018). Chloroquine inhibits autophagic flux by decreasing autophagosome-lysosome fusion. Autophagy.

[11] Huss M., Ingenhorst G., König S., Gassel M., Dröse S., Zeeck A. (2002). Concanamycin A, the specific inhibitor of V-ATPases, binds to the V(o) subunit c. J. Biol. Chem..

[12] Höhn A., Grune T. (2013). Lipofuscin: formation, effects and role of macroautophagy. Redox Biol..

[13] Lipofuscin Wolf G. (1993). The age pigment. Nutr. Rev..

[14] Kakimoto Y., Okada C., Kawabe N., Sasaki A., Tsukamoto H., Nagao R. (2019). Myocardial lipofuscin accumulation in ageing and sudden cardiac death. Sci. Rep..

[15] Baldensperger T., Jung T., Heinze T., Schwerdtle T., Höhn A., Grune T. (2024). The age pigment lipofuscin causes oxidative stress, lysosomal dysfunction, and pyroptotic cell death. Free Radic. Biol. Med..

[16] Höhn A., Sittig A., Jung T., Grimm S., Grune T. (2012). Lipofuscin is formed independently of macroautophagy and lysosomal activity in stress-induced prematurely senescent human fibroblasts. Free Radic. Biol. Med..

[17] Terman A., Abrahamsson N., Brunk U.T. (1999). Ceroid/lipofuscin-loaded human fibroblasts show increased susceptibility to oxidative stress. Exp. Gerontol..

[18] Höhn A., Jung T., Grimm S., Catalgol B., Weber D., Grune T. (2011). Lipofuscin inhibits the proteasome by binding to surface motifs. Free Radic. Biol. Med..

[19] Li W.-W., Wang H.-J., Tan Y.-Z., Wang Y.-L., Yu S.-N., Li Z.-H. (2021). Reducing lipofuscin accumulation and cardiomyocytic senescence of aging heart by enhancing autophagy. Exp. Cell Res..

[20] Porta E., Llesuy S., Monserrat A.J., Benavides S., Travacio M. (1995). Changes in cathepsin B and lipofuscin during development and aging in rat brain and heart. Gerontology.

[21] Liang W., Moyzis A.G., Lampert M.A., Diao R.Y., Najor R.H., Gustafsson Å.B. (2020). Aging is associated with a decline in Atg9b-mediated autophagosome formation and appearance of enlarged mitochondria in the heart. Aging Cell.

[22] Lim C.C., Apstein C.S., Colucci W.S., Liao R. (2000). Impaired cell shortening and relengthening with increased pacing frequency are intrinsic to the senescent mouse cardiomyocyte. J. Mol. Cell. Cardiol..

[23] Grandy S.A., Howlett S.E. (2006). Cardiac excitation-contraction coupling is altered in myocytes from aged male mice but not in cells from aged female mice. Am. J. Physiol. Heart Circ. Physiol..

[24] Domeier T.L., Roberts C.J., Gibson A.K., Hanft L.M., McDonald K.S., Segal S.S. (2014). Dantrolene suppresses spontaneous Ca2+ release without altering excitation-contraction coupling in cardiomyocytes of aged mice. Am. J. Physiol. Heart Circ. Physiol..

[25] Höhn A., Jung T., Grimm S., Grune T. (2010). Lipofuscin-bound iron is a major intracellular source of oxidants: role in senescent cells. Free Radic. Biol. Med..

[26] Ackers-Johnson M., Li P.Y., Holmes A.P., O'Brien S.-M., Pavlovic D., Foo R.S. (2016). A simplified, langendorff-free method for concomitant isolation of viable cardiac myocytes and nonmyocytes from the adult mouse heart. Circ. Res..

[27] Walter S., Jung T., Herpich C., Norman K., Pivovarova-Ramich O., Ott C. (2023). Determination of the autophagic flux in murine and human peripheral blood mononuclear cells. Front. Cell Dev. Biol..

[28] Bauer B., Martens S., Ferrari L. (2023). Aggrephagy at a glance. J. Cell Sci..

[29] Feridooni H.A., Dibb K.M., Howlett S.E. (2015). How cardiomyocyte excitation, calcium release and contraction become altered with age. J. Mol. Cell. Cardiol..

[30] König J., Ott C., Hugo M., Jung T., Bulteau A.-L., Grune T. (2017). Mitochondrial contribution to lipofuscin formation. Redox Biol..

[31] Sohal R.S., Marzabadi M.R., Galaris D., Brunk U.T. (1989). Effect of ambient oxygen concentration on lipofuscin accumulation in cultured rat heart myocytes--a novel in vitro model of lipofuscinogenesis. Free Radic. Biol. Med..

[32] McElnea E.M., Hughes E., McGoldrick A., McCann A., Quill B., Docherty N. (2014). Lipofuscin accumulation and autophagy in glaucomatous human lamina cribrosa cells. BMC Ophthalmol..

[33] Ko J., Jang Y.C., Quindry J., Guttmann R., Cosio-Lima L., Powers S.K. (2023). Exercise-induced antisenescence and autophagy restoration mitigate metabolic disorder-induced cardiac disruption in mice. Med. Sci. Sports Exerc..

[34] Myeku N., Figueiredo-Pereira M.E. (2011). Dynamics of the degradation of ubiquitinated proteins by proteasomes and autophagy: association with sequestosome 1/p62. J. Biol. Chem..

[35] Tatti M., Motta M., Di Bartolomeo S., Scarpa S., Cianfanelli V., Cecconi F. (2012). Reduced cathepsins B and D cause impaired autophagic degradation that can be almost completely restored by overexpression of these two proteases in Sap C-deficient fibroblasts. Hum. Mol. Genet..

[36] Song S.B., Shim W., Hwang E.S. (2023). Lipofuscin granule accumulation requires autophagy activation. Mol. Cells.

[37] Lamming D.W. (2016). Inhibition of the mechanistic target of rapamycin (mTOR)-Rapamycin and beyond. Cold Spring Harb Perspect Med.

[38] Fernández Á.F., Sebti S., Wei Y., Zou Z., Shi M., McMillan K.L. (2018). Disruption of the beclin 1-BCL2 autophagy regulatory complex promotes longevity in mice. Nature.

[39] Guo R., Hu N., Kandadi M.R., Ren J. (2012). Facilitated ethanol metabolism promotes cardiomyocyte contractile dysfunction through autophagy in murine hearts. Autophagy.

[40] Peng H., Zhang J., Zhang Z., Turdi S., Han X., Liu Q. (2023). Cardiac-specific overexpression of catalase attenuates lipopolysaccharide-induced cardiac anomalies through reconciliation of autophagy and ferroptosis. Life Sci..

[41] Jaber N., Dou Z., Chen J.-S., Catanzaro J., Jiang Y.-P., Ballou L.M. (2012). Class III PI3K Vps34 plays an essential role in autophagy and in heart and liver function. Proc. Natl. Acad. Sci. U. S. A..

[42] Yano K., Yanagisawa T., Mukae K., Niwa Y., Inoue Y., Moriyasu Y. (2015). Dissection of autophagy in tobacco BY-2 cells under sucrose starvation conditions using the vacuolar H(+)-ATPase inhibitor concanamycin A and the autophagy-related protein Atg8. Plant Signal. Behav..

[43] Tanaka Y., Guhde G., Suter A., Eskelinen E.L., Hartmann D., Lüllmann-Rauch R. (2000). Accumulation of autophagic vacuoles and cardiomyopathy in LAMP-2-deficient mice. Nature.

[44] Mubagwa K. (2020). Cardiac effects and toxicity of chloroquine: a short update. Int. J. Antimicrob. Agents.

[45] Chi C., Leonard A., Knight W.E., Beussman K.M., Zhao Y., Cao Y. (2019). LAMP-2B regulates human cardiomyocyte function by mediating autophagosome-lysosome fusion. Proc. Natl. Acad. Sci. U. S. A..

[46] Alcalai R., Arad M., Wakimoto H., Yadin D., Gorham J., Wang L. (2021). LAMP2 cardiomyopathy: consequences of impaired autophagy in the heart. J. Am. Heart Assoc..

[47] Christensen K.A., Myers J.T., Swanson J.A. (2002). pH-dependent regulation of lysosomal calcium in macrophages. J. Cell Sci..

[48] Aston D., Capel R.A., Ford K.L., Christian H.C., Mirams G.R., Rog-Zielinska E.A. (2017). High resolution structural evidence suggests the Sarcoplasmic Reticulum forms microdomains with Acidic Stores (lysosomes) in the heart. Sci. Rep..

[49] Xie an, Kang G.-J., Kim E.J., Feng F., Givens S.E., Ogle B.M. (2023). Lysosomal Ca2+ flux modulates automaticity in ventricular cardiomyocytes and correlates with arrhythmic risk. PNAS Nexus.

[50] Farombi E.O. (2006). Genotoxicity of chloroquine in rat liver cells: protective role of free radical scavengers. Cell Biol. Toxicol..

[51] Xu K.Y., Zweier J.L., Becker L.C. (1997). Hydroxyl radical inhibits sarcoplasmic reticulum Ca(2+)-ATPase function by direct attack on the ATP binding site. Circ. Res..

[52] Hafstad A.D., Nabeebaccus A.A., Shah A.M. (2013). Novel aspects of ROS signalling in heart failure. Basic Res. Cardiol..

[53] Redmann M., Benavides G.A., Berryhill T.F., Wani W.Y., Ouyang X., Johnson M.S. (2017). Inhibition of autophagy with bafilomycin and chloroquine decreases mitochondrial quality and bioenergetic function in primary neurons. Redox Biol..

