# Supplementary material for: Oxidized protein aggregate lipofuscin impairs cardiomyocyte contractility via late-stage autophagy inhibition
Source: Redox Biol. 2025 Feb 19;81:103559. doi: 10.1016/j.redox.2025.103559 (PMC11938141; doi:10.1016/j.redox.2025.103559)
Supplement: Multimedia component 1 [file mmc1.docx]

**Supplementary Material**

**Supplements Method: Viability assay**

To test for viability, cells were plated in 96-well plates and treated with 0.05 mg/ml lipofuscin in PBS. PBS was used as a solvent control. After 24 h, 3-[4,5-dimethylthiazol-2-yl]-2,5 diphenyl tetrazolium bromide (MTT) was added to a final concentration of 0.83 mg/ml to each well. After incubation for 40 min at 37°C, the supernatant was discarded and 100 µl 5% formic acid in isopropanol were added to each well. The plate was shaken for 10 min followed by a photometric measurement at 550 nm with a reference wavelength of 690 nm.


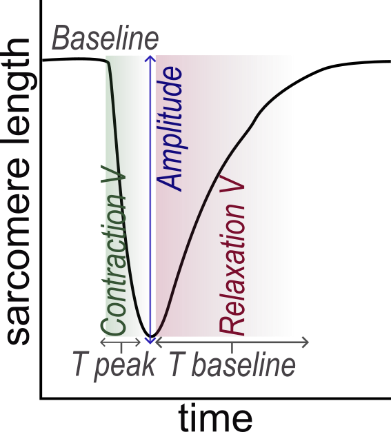


**Supplements Figure 1: Cardiomyocyte contraction parameters.** Contraction transients were analyzed by changes in sarcomere length per time. Amplitude was calculated by dividing peak height by baseline. V=velocity. T=time.

**Supplements Figure 2: Effect of lipofuscin on cardiomyocyte viability.** Young cardiomyocytes were treated for 18 h with 0.05 mg/ml lipofuscin and viability was analyzed using 3-[4,5-dimethylthiazol-2-yl]-2,5 diphenyl tetrazolium bromide (MTT). Data represent mean ± SD. One sample t-test did not reveal statistical significance.

**c**

**b**

**a**

**e**

**d**

**Supplements Figure 3: Effect of cultivation on cardiomyocyte contraction.** Contraction of young control cardiomyocytes was observed over a period of 2 to 18 h. Data represent mean ± SD. Statistical significance was tested with One-way ANOVA and given as follows: *p≤0.05, **p≤0.01, ***p≤0.001.


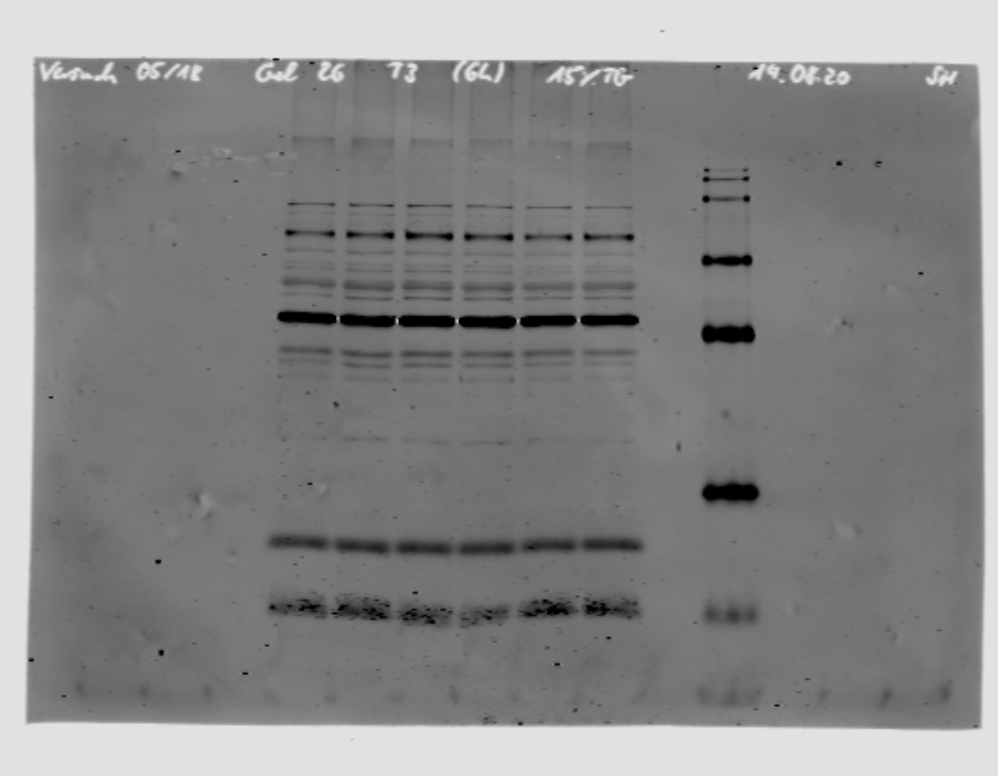

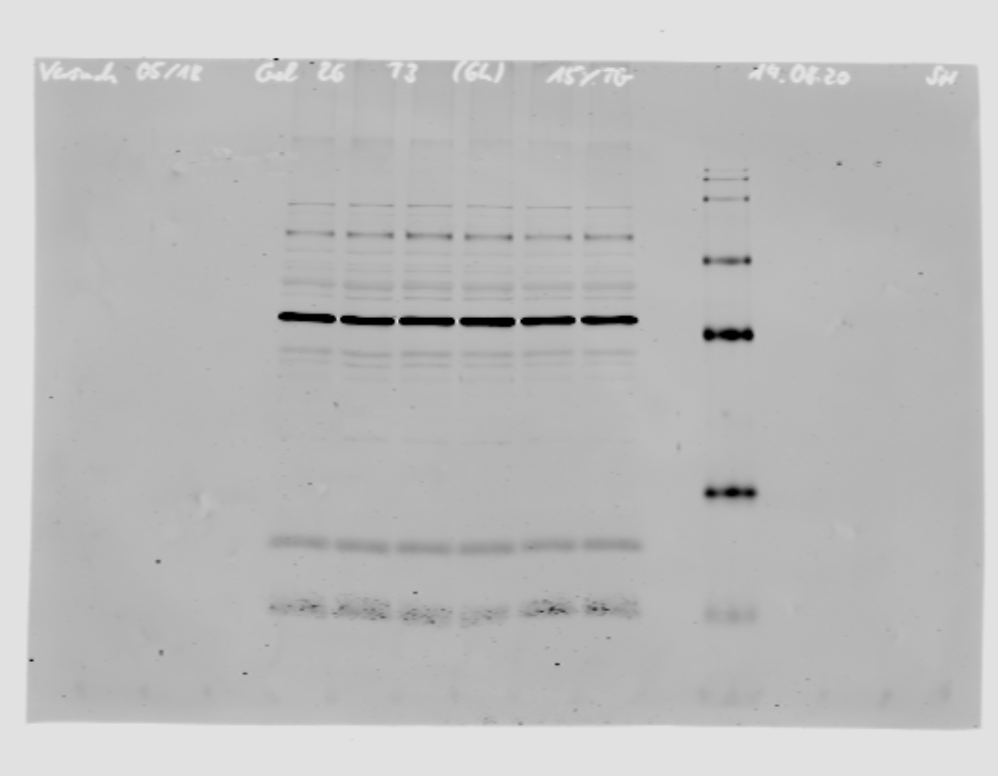


p62

GAPDH

Lipo 6 h

PBS 6 h

**b**

**a**

**Supplements Figure 4: Effect of 6 h lipofuscin treatment on p62.** Primary cardiomyocytes were treated for 6 h with 0.05 mg/ml lipofuscin or PBS as a solvent control. Protein levels of p62 were evaluated via immunoblot using GAPDH as a housekeeping protein. Unpaired t-test did not reveal statistical significance.

**a**

**c**

**b**

**Supplements Figure 5: Effect of 2 h CQ treatment on cardiomyocyte contraction.** Primary cardiomyocytes were treated for 2 h with the late-stage autophagic flux inhibitor chloroquine (CQ). Unpaired t-test did not reveal statistical significance.

**Supplements Figure 6: Effect of autophagic flux inhibitors on LC3-I protein levels.** Primary cardiomyocytes were treated for 24 h with autophagic flux inhibitors 3-methyladenine (3-MA), chloroquine (CQ) and concanamycin A (ConA). Induction of LC3-I protein levels were analyzed using immunoblot. Data represent mean ± SD. Statistical significance was tested with one sample t-test (normality passed) or one sample Wilcoxon test (normality not passed) and given as follows: ***p≤0.001, ****p≤0.0001.
